# Inhibition of micro RNA miR-122-5p prevents lipopolysaccharide-induced myocardial injury by inhibiting oxidative stress, inflammation and apoptosis via targeting GIT1

**DOI:** 10.1080/21655979.2021.1926201

**Published:** 2021-05-18

**Authors:** Wenliang Song, Tiening Zhang, Ni Yang, Tao Zhang, Ri Wen, Chunfeng Liu

**Affiliations:** Department of Pediatrics, PICU, Shengjing Hospital of China Medical University, Shenyang, Liaoning Province, China

**Keywords:** miR-122-5p, GIT1, sepsis, myocardial injury

## Abstract

Myocardial injury resulting from sepsis is the leading cause of death worldwide. Micro RNA miR-122-5p is involved in various physiological and pathological processes and is highly expressed in the heart of septic rats. However, its function in sepsis-caused myocardial injury remains elusive. Herein, a rat model of septic myocardial injury was established by intraperitoneal injection of lipopolysaccharide (LPS), and cardiomyocyte H9c2 was exposed to LPS to induce sepsis-related inflammatory injury *in vitro*. Inhibition of miR-122-5p suppressed LPS-triggered myocardial injury evidenced by decreased heart weight index (HWI), reduced inflammatory cell infiltration and cell rupture, and reduced cardiac marker enzymes cTnI and LDH. MiR-122-5p inhibition inhibited ROS production and enhanced the activities of antioxidant enzymes CAT, SOD and GSH-px in LPS-treated rats and H9c2 cells. MiR-122-5p inhibition reduced the production of pro-inflammatory cytokines TNF-α, IL-6 and IL-1β, and inhibited cell apoptosis along with decreased cleaved-caspase 3 induced by LPS. Moreover, increased GIT1 expression was found following miR-122-5p inhibition. We further verified GIT1 as a target of miR-122-5p, and silencing GIT1 partially reversed the benefits of miR-122-5p loss in LPS-injured H9c2 cells. The HO-1 and NQO-1 expression and Nrf-2 activation were enhanced by miR-122-5p inhibition, which was reversed by GIT1 depletion, indicating the involvement of Nrf-2/HO-1 signaling in regulating miR-122-5p/GIT1-mediated cardioprotection. Taken together, our data suggest that inhibition of miR-122-5p may mitigate sepsis-triggered myocardial injury through inhibiting inflammation, oxidative stress and apoptosis via targeting GIT1, which provides a possible therapeutic target for sepsis.

## GRAPHICAL ABSTRACT

**Figure:**
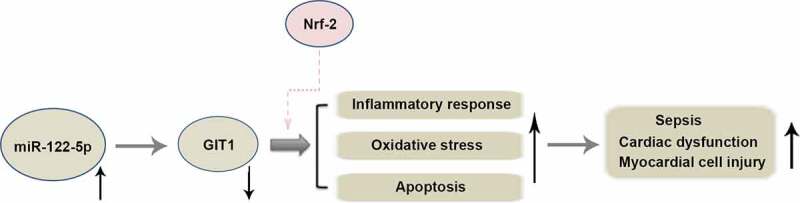

## 1. Introduction

Sepsis is a life-threatening syndrome resulted from abnormal systemic host response to infection [1]. Despite significant advances in the prevention and treatment of sepsis through the use of appropriate antibiotics, resuscitation with intravenous fluids and vasoactive drugs, sepsis remains a major healthcare challenge [2,3]. Recently, the occurrence and pathogenesis of sepsis are closely linked to excessive inflammation and failure of immune system that ultimately contributes to cell death [4,5]. Effective prevention of inflammatory response may alleviate sepsis injury. Approximately 40% to 60% of the patients suffer from sepsis with heart, kidney, brain and other organ failure [6,7]. Myocardial injury is the most common clinical manifestation of sepsis [8]. Hence, it is essential to identify new therapeutic strategies to restore cardiac function for declining the mortality in patients with sepsis.

MicroRNAs (miRNAs) are endogenous RNA molecules of 18–25 nucleotides that play key roles in development, aging, and cell death [9,10]. It is reported that miRNAs can bind to the 3ʹuntranslated region (3ʹUTR) of protein-coding genes to regulate their expression at the post-transcriptional level [11,12]. Recently, several miRNAs have been proven to be involved in the development of sepsis. Inhibition of miR-21-3p or elevation of miR-146a can alleviate sepsis-induced myocardial depression by reducing inflammatory response [13,14]. MiR-150-5p reduces myocardial depression in sepsis by inhibiting cardiomyocyte apoptosis [15]. Importantly, miR-122-5p is highly expressed in septic extracellular vesicles and lipopolysaccharide (LPS)-induced septic rats [16,17]. Knockdown of miR-122-5p inhibits macrophage-related inflammatory responses [18]. However, the role of miR-122-5p in sepsis-induced myocardial injury and its underlying mechanism are not yet fully understood.

G-protein-coupled receptor kinase interacting protein-1 (GIT1) is a scaffold protein containing various functional domains that possesses anti-inflammatory and anti-apoptotic activities [19]. Upregulation of GIT1 represses chondrocyte apoptosis [20]. GIT1 deletion increases the generation of pro-inflammatory cytokine interleukin 1β (IL-1β) in LPS-activated macrophages [21]. Besides, GIT1 plays a critical role in heart. GIT1 knockdown results in the increase of cardiomyocyte apoptosis and aggravates myocardial injury [22]. Bioinformatics prediction suggested that miR-122-5p may bind to the 3ʹUTR of GIT1. Therefore, we hypothesize that miR-122-5p exerts its function via modulation of GIT1.

In our current study, to explore the function of miR-122-5p in LPS-triggered myocardial injury and its potential mechanism, we constructed both *in vivo* and *in vitro* models of sepsis by LPS challenge. MiR-122-5p expression was remarkably increased and GIT1 was reduced in heart tissues and H9c2 cardiomyocytes in response to LPS. Moreover, further experiments demonstrated the cardioprotective role of miR-122-5p inhibition in sepsis, which was the first to reveal the effect of miR-122-5p on sepsis-induced myocardial injury. Our findings suggest that miR-122-5p may be a promising therapeutic target for myocardial injury post sepsis.

## 2. Materials and methods

### 2.1. Animal care and sepsis model

Healthy male Wistar rats (170–190 g, obtained from Liaoning Changsheng Biotechnology Co., Ltd., Benxi, China) were housed in a controlled condition with a temperature of 22 ± 1°C, a humidity of 45–55% and a programmed 12-h light/12-h dark cycle for circadian control. All procedures carried out in our study were in accordance with the protocols of ethical committees of Shengjing Hospital of China Medical University (2019PS073K) for the Animal Care and Use.

After 1 week of acclimatization, the rats were divided into four groups, including control, LPS, LPS + negative control antagomir (NC antagomir), LPS + miR-122-5p antagomir groups (n = 6 rats per group). Each rat was anesthetized by 20% urethane. Septic myocardial injury in rats was induced by intraperitoneal injection of LPS (20 mg/kg) establishing a septic shock model as described previously [17,23]. The control rats were given the equal volumes of saline. Prior to this, the experimental rats were injected via tail vein with 50 nmol/kg miR-122-5p antagomir or NC antagomir for 24 h [24]. Twelve hours after LPS stimulation, the rats were euthanized and weighted (body weight, BW). The heart was excised and immediately weighted (heart weight, HW). The heart weight index (HWI) was calculated as HWI = HW/BW [25]. After that, the heart tissues were harvested; some were flash frozen in lipid nitrogen and some were fixed with 4% paraformaldehyde.

### 2.2. Histological staining

Heart tissues were fixed with 4% paraformaldehyde and permeabilized with xylene. After paraffin-embedding and dewaxing, the sections were stained with hematoxylin solution and eosin solution (H&E; Solarbio, Beijing, China; Sangon, Shanghai, China). Finally, the pathological changes were visualized using a microscope under 200× magnification.

### 2.3. Cell culture and treatments

Rat H9c2 cardiomyocytes were selected to explore the role of miR-122-5p in our *in vitro* study. H9c2 cells were purchased from the Shanghai Institute of Biochemistry and Cell Biology (Shanghai, China) and maintained in DMEM medium supplemented with 10% fetal bovine serum (FBS).

When the cell density reached 70%, part of H9c2 cells were transfected with miR-122-5p inhibitor or NC inhibitor. The other cells were co-transfected with miR-122-5p inhibitor and GIT1 siRNA or NC siRNA. Cell transfection was performed using lipofectamine 3000 reagent according to the protocol of manufacture. The sequences of GIT1 siRNA are 5ʹ-GCCAGGCCUUCUCUAUGUATT-3ʹ and 5ʹ-UACAUAGAGAAGGCCUGGCTT-3ʹ. After 24 h of transfection, H9c2 cells were incubated with 10 µg/ml LPS [26] for 24 h to induce *in vitro* sepsis model and then harvested for subsequent experiments.

### 2.4. Biochemistry analysis

The concentrations of cardiac troponin I (cTnI) and lactate dehydrogenase (LDH) were determined following the procedures of the respective detection kits. Besides, the production of reactive oxygen species (ROS), the activities of enzymes catalase (CAT), superoxide dismutase (SOD), glutathione peroxidase (GSH-px), and the levels of inflammatory cytokines tumor necrosis factor alpha (TNF-α), interleukin-6 (IL-6) and IL-1β were detected using the corresponding commercial kits. The kits for determining cTnI, TNF-α, IL-6 and IL-1β levels were purchased from USCN KIT INC. (Wuhan, China), and the other kits were obtained from Nanjing Jiancheng Bioengineering Institute (Nanjing, China).

### 2.5. Reverse transcription real-time quantitative PCR (RT-qPCR)

Total RNA from heart tissues or H9c2 cells was isolated using a corresponding extraction kit and then reverse-transcribed into cDNA. Real-time quantitative PCR (RT-qPCR) was performed to detect gene expression levels using a 7500 Real-Time PCR System (Applied Biosystems, Waltham, MA, USA) according to TB Green® Premix Ex Taq™ II (Tli RNase H Plus) kit instructions (TaKaRa, Tokyo, Japan). GAPDH, Histone H3 or U6 was selected for normalization. Primer sequences, synthesized by Sangon Biotech (Shanghai, China), are shown below: rno-miR-122-5p forward, 5ʹ-CGTGGAGTGTGACAATGGTGTT-3ʹ and reverse, 5ʹ-GTGCAGGGTCCGAGGTATTC-3ʹ; U6 forward, 5ʹ-CTCGCTTCGGCAGCACA-3ʹ and reverse 5ʹ-AACGCTTCACGAATTTGCGT-3ʹ; GIT1 forward, 5ʹ-AGTGAACGGGCAGAACA-3ʹ and reverse 5ʹ-GAGGAGGGAGTGAAGGTC-3ʹ; heme oxygenase-1 (HO-1) forward, 5ʹ- CGAAACAAGCAGAACCCA-3ʹ and reverse 5ʹ-CACCAGCAGCTCAGGATG-3ʹ; NAD(P)H: quinone oxidoreductase 1 (NQO-1) forward, 5ʹ-GTATGCCACCATGTATGAC-3ʹ and reverse 5ʹ- GCTTGGAGCAAAGTAGAG-3ʹ; GAPDH forward, 5ʹ-CGGCAAGTTCAACGGCACAG-3ʹ and reverse 5ʹ-CGCCAGTAGACTCCACGACAT-3ʹ.

### 2.6. Western blot analysis

Total protein was extracted using RIPA solution, and quantified with BCA protein assay kit (Beyotime, Shanghai, China). Protein samples (20 µg) were then subjected to electrophoresis on SDS-PAGE gels (Solarbio, Beijing, China) before transferring onto PVDF membranes (Millipore, USA). After blocking with 5% nonfat milk for 1 h, the proteins were incubated overnight at 4°C with corresponding primary antibodies against GIT1 (1: 500; Boster, USA), caspase 3 (1: 1000; CST, USA), nuclear factor erythroid 2-related factor 2 (Nrf-2; 1: 500; Affinity, China) and GAPDH (1: 10000; Proteintech, China). Then, the PVDF membranes were washed with TBST, followed by incubation for 1 h with anti-rabbit or anti-mouse IgG-peroxidase secondary antibody (1: 3000) at room temperature. Protein bands were visualized using an enhanced chemiluminescence (ECL) system and analyzed by Gel-Pro-Analyzer software.

### 2.7. Terminal deoxynucleotidyl transferase dUTP nick end labeling (TUNEL) staining

Cardiac apoptosis was examined using a cell death detection kit (Roche, Switzerland) according to the manufacturer’s recommended procedure. The heart samples were fixed, paraffin-embedded, sectioned (5 μm) and then dewaxed. After permeabilization with 0.1% Triton X–100 for 8 min, the sections were labeled with TUNEL reagent and then counterstained with DAPI in the dark. All the fluorescent digital photographs were taken under a 400 × microscope.

### 2.8. Flow cytometric analysis of cell apoptosis and ROS generation

Apoptosis of H9c2 cells was determined by a cell apoptosis detection kit (Dojindo, Japan). After centrifugation at 1000 g for 5 min, the cells were resuspended in the binding buffer and then treated with 5 μl Annexin V-FITC reagent and 5 μl Propidium Iodide (PI) in the darkness (4°C, 15 min). The apoptosis rate was evaluated using a flow cytometer.

The ROS content in H9c2 cells was detected by the ROS detection kit (Keygen, Nanjing, China). H9c2 cells were centrifuged at 140 g for 5 min and incubated with 1 ml DCFH-DA for 30 min (37°C). After resuspension in 500 μl PBS, flow cytometry was performed to measure the ROS content.

### 2.9. Luciferase reporter assay

The wild-type (WT) and mutant (Mut) 3ʹUTR sequences of GIT1 were synthesized in Nanjing Genscript Co., ltd. (Nanjing, China) and inserted into luciferase reporter vectors, and named as GIT1 3ʹUTR (WT) and GIT1 3ʹUTR (Mut), respectively. After that, H9c2 cells were co-transfected with the recombinant plasmid and miR-122-5p mimics or NC mimics using lipofectamine 3000. After 48 h, luciferase activity was measured using the dual-luciferase reporter assay system (Keygen, Nanjing, China). The luciferase activity value was normalized with Renilla luciferase control.

### 2.10. Statistical analysis

All statistical calculations and analyses were performed using the GraphPad Prism 8.0 software. Statistical differences between two groups were analyzed by student’s *t*-test, while comparison between multiple groups was analyzed using one-way analysis of variance (ANOVA) followed by Tukey’s multiple comparisons test. The results were expressed as mean ± SD. The difference was considered significant with a value of *p* < 0.05.

## 3. Results

### 3.1. Inhibition of miR-122-5p alleviates LPS-induced myocardial injury in rats

**Figure 1.**
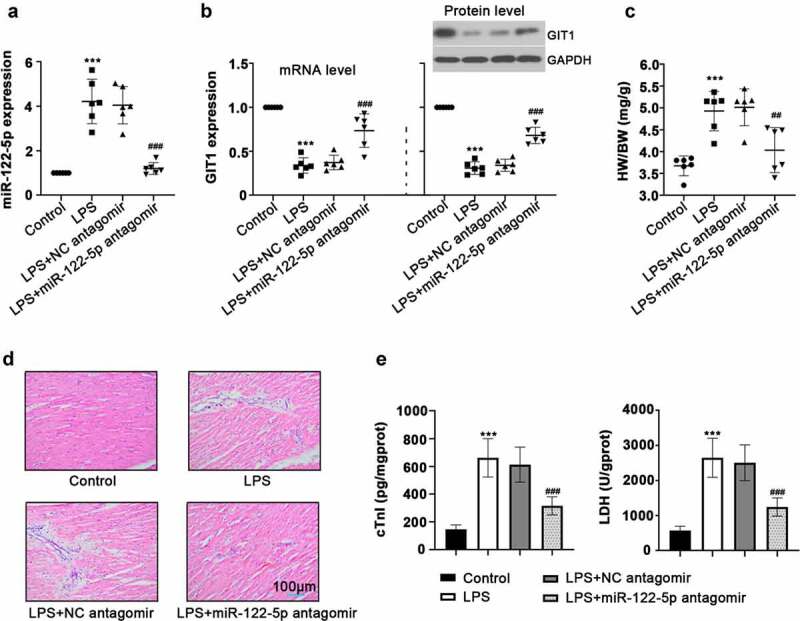
Inhibition of micro RNA miR-122-5p on lipopolysaccharide-induced myocardial injury. Wistar rats were intravenously injected with miR-122-5p antagomir, followed by lipopolysaccharide (LPS) stimulation (n = 6 rats per group). (a-b) After LPS treatment for 12 h, heart tissues were harvested for the relative expression levels of miR-122-5p and G-protein-coupled receptor kinase interacting protein-1 (GIT1) using real-time quantitative PCR (RT-qPCR) or western blot assay. (c) The ratio of heart weight/body weight (HW/BW) was calculated. (d) Hematein and eosin (H&E) staining revealed the effect of miR-122-5p on LPS-induced histopathological changes in heart. (e) Levels of cardiac troponin I (cTnI) and lactate dehydrogenase (LDH) were examined by enzyme-linked immunosorbent assay (ELISA). ***p < 0.001 versus control; ^##^*p* < 0.01, ^###^*p* < 0.001 versus LPS + NC antagomir

To establish a sepsis model *in vivo*, the rats were intraperitoneally injected with 20 mg/kg LPS. As illustrated in Figure 1, at 12 h after LPS treatment, we observed increased miR-122-5p expression and reduced GIT1 mRNA and protein levels. Moreover, HW/BW was greater in the LPS-induced rats than that in the control group. H&E staining results showed that LPS triggered infiltration and rupture of inflammatory cells in the heart. Biochemical analysis revealed that LPS induced increased levels of cTnI and LDH, markers of myocardial injury. These data indicated that a sepsis model was successfully induced in rats.

To study the function of miR-122-5p in sepsis-induced heart injury, the rats were administrated with miR-122-5p antagomir or NC antagomir, followed by sepsis induction. Twelve hours after LPS treatment, miR-122-5p expression was significantly down-regulated but GIT1 was up-regulated by miR-122-5p antagomir injection (Figure 1(a,b)). Moreover, miR-122-5p inhibition reduced the HWI of septic rats compared with the LPS+NC antagomir group (Figure 1(c)). H&E staining suggested that miR-12-5p inhibition apparently improved LPS-caused inflammatory damage in heart (Figure 1(d)). Inhibition of miR-122-5p lowered the levels of cTnI and LDH in the heart (Figure 1(e)). These results indicate that miR-122-5p inhibition has a protective effect on LPS-induced myocardial injury, which may be related to the regulation of GIT1.

### 3.2. Inhibition of miR-122-5p attenuates oxidative stress, inflammation and apoptosis in sepsis rats

**Figure 2.**
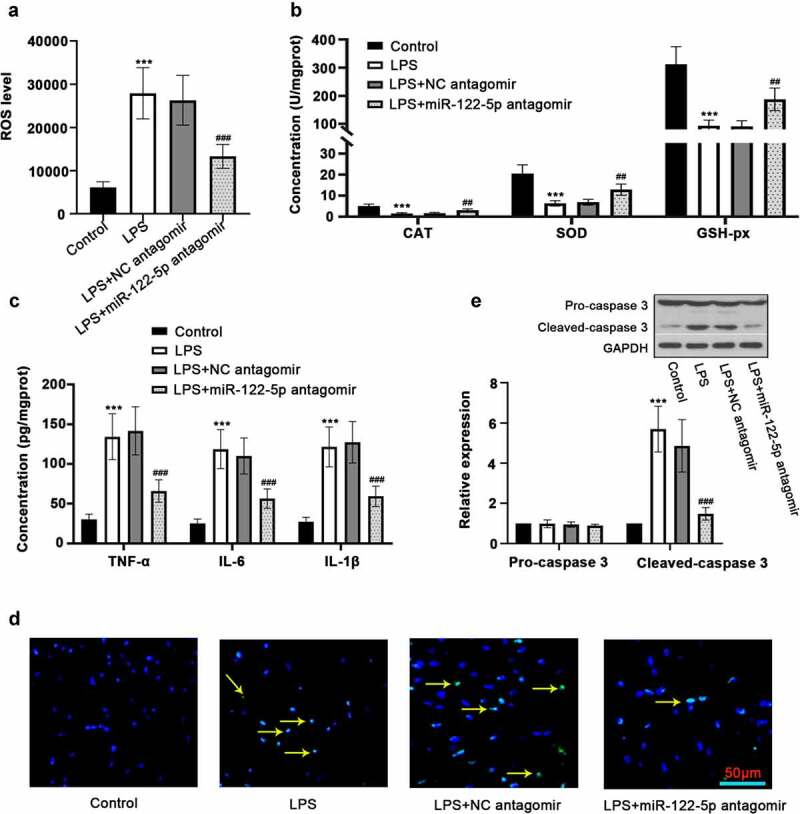
Inhibition of micro RNA miR-122-5p prevents myocardial injury in sepsis rats. (a) The production of reactive oxygen species (ROS) in the heart was determined using enzyme-linked immunosorbent assay (ELISA). (b) The activities of catalase (CAT), superoxide dismutase (SOD) and glutathione peroxidase (GSH-px), the key enzymes related to oxidative stress, were measured. (c) The release of inflammatory cytokines tumor necrosis factor alpha (TNF-α), interleukin-6 (IL-6) and IL-1β was analyzed by ELISA kits. (d) Hearts were sectioned for terminal deoxynucleotidyl transferase dUTP nick end labeling (TUNEL) analysis to visualize lipopolysaccharide (LPS)-triggered apoptosis. (e) Western blot was carried out for determining the expression of caspase-3, a protein associated with apoptosis. ***p < 0.001 versus control; ^##^*p* < 0.01, ^###^*p* < 0.001 versus LPS + NC antagomir

To clarify the regulatory mechanisms of miR-122-5p in sepsis-induced myocardial injury, the following experiments were conducted. Based on biochemistry analysis, we noticed that the relative ROS level in the LPS group was significantly higher than that in the control group, while it was decreased by miR-122-5p inhibition (Figure 2(a)). The activities of CAT, SOD and GSH-px were decreased in the heart of septic rats, while inhibiting miR-122-5p increased their activities (Figure 2(b)). Moreover, LPS strongly augmented the release of pro-inflammatory factors TNF-α, IL-6, and IL-1β, which could be partially reversed by inhibition of miR-122-5p (Figure 2(c)). In the sepsis group, there were a large number of TUNEL-positive cells, whereas miR-122-5p inhibition reduced the degree of apoptosis in the heart (Figure 2(d)). Western blot analysis further confirmed that miR-122-5p inhibition suppressed cell apoptosis evidenced by down-regulated cleaved-caspase 3 expression (Figure 2(e)). These results suggest that miR-122-5p inhibition ameliorates LPS-induced oxidative stress, inflammatory response and apoptosis in heart of rats.

### 3.3. Inhibition of miR-122-5p repressed LPS-induced cell damage

**Figure 3.**
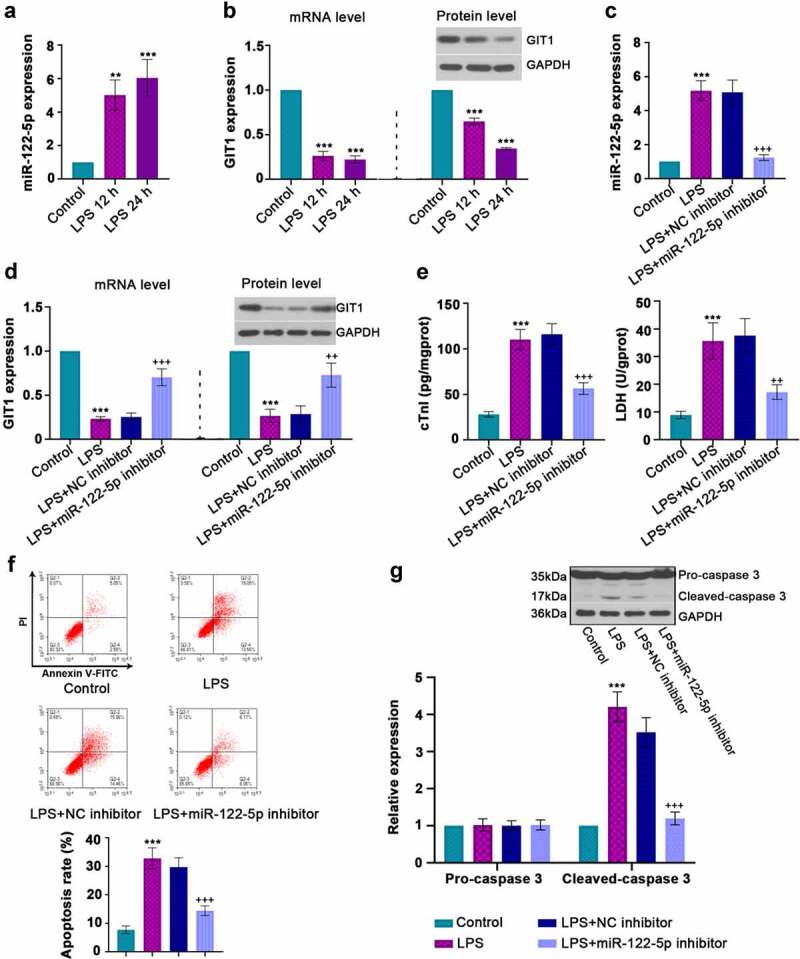
*In vitro* analysis for beneficial role of inhibiting micro RNA miR-122-5p in lipopolysaccharide (LPS)-induced apoptosis. (a-b) Rat H9c2 cells were treated with LPS for 12 h or 24 h, and the expression levels of miR-122-5p and G-protein-coupled receptor kinase interacting protein-1 (GIT1) were assessed by real-time quantitative PCR (RT-qPCR) or western blot analysis. (c-d) H9c2 cells were transfected with NC inhibitor or miR-122-5p inhibitor for 24 h, followed by LPS treatment for another 24 h under proper culture conditions. After that, miR-122-5p and GIT1 expression levels were measured. (e) The contents of cardiac troponin I (cTnI) and lactate dehydrogenase (LDH) were detected by appropriate kits. (f) Flow cytometry showed the apoptosis of LPS-stimulated myocardial cells. (g) Western blot analysis illustrated the changes of caspase-3 expression. **p < 0.01, ***p < 0.001 versus control; ^++^*p* < 0.01, ^+++^*p* < 0.001 versus LPS + NC inhibitor

**Figure 4.**
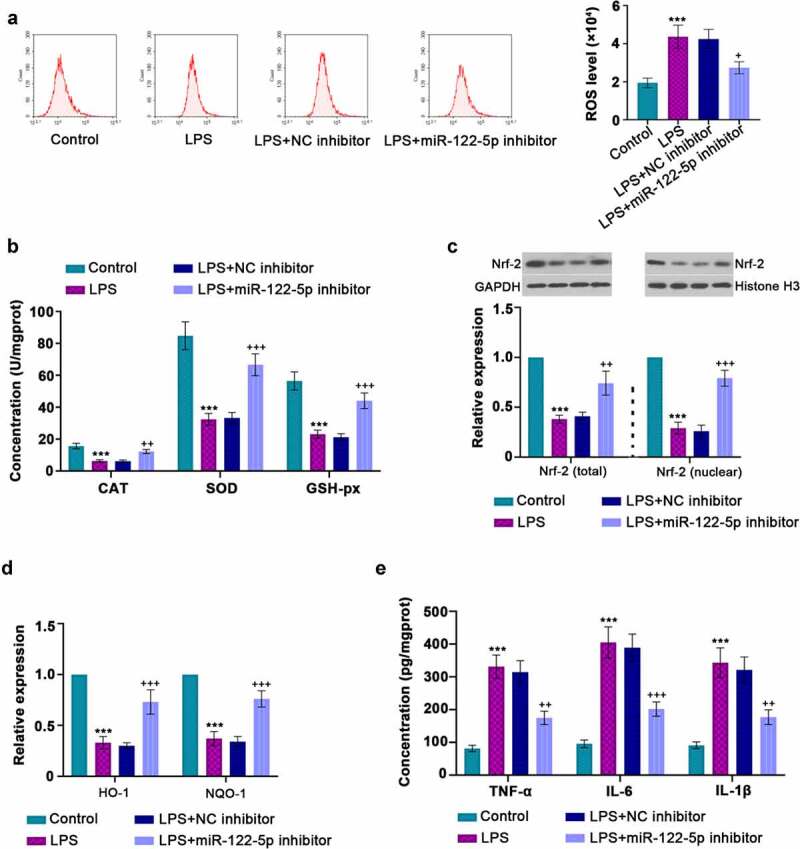
Inhibition of micro RNA miR-122-5p suppresses lipopolysaccharide (LPS)-induced oxidative stress and inflammatory response. (a) Reactive oxygen species (ROS) production was determined by a flow cytometer. (b) The enzymes catalase (CAT), superoxide dismutase (SOD), glutathione peroxidase (GSH-px) activities were measured using the manufacturer’s kits. (c) Western blot was performed to measure the nuclear factor erythroid 2-related factor 2 (Nrf-2) expression in the cytoplasm and nucleus. (d) Real-time quantitative PCR (RT-qPCR) was used for heme oxygenase-1 (HO-1) and NAD(p)H: quinone oxidoreductase 1 (NQO-1) levels. (e) The concentrations of tumor necrosis factor alpha (TNF-α), interleukin-6 (IL-6) and IL-1β were quantified by enzyme-linked immunosorbent assay (ELISA) kits. ***p < 0.001 versus control; ^+^*p* < 0.05, ^++^*p* < 0.01, ^+++^*p* < 0.001 versus LPS + NC inhibitor

To induce inflammatory injury *in vitro*, H9c2 cells were stimulated with 10 µg/ml LPS. It was shown that LPS treatment increased miR-122-5p expression but decreased GIT1 in a time-dependent manner in H9c2 cardiomyocytes (Figure 3(a,b)). To further validate the role of miR-122-5p in sepsis injury, H9c2 cells were transfected with miR-122-5p inhibitor or NC inhibitor, followed by LPS exposure. After 24 h of sepsis induction, RT-qPCR analysis demonstrated that miR-122-5p inhibitor transfection induced the decrease in miR-122-5p expression and the increase in GIT1 mRNA and protein levels (Figure 3(c,d)). Besides, miR-122-5p inhibition reduced the concentrations of cTnI and LDH (Figure 3(e)). Inhibiting miR-122-5p diminished LPS-triggered cell apoptosis, along with the reduction of cleaved-caspase 3 expression (Figure 3(f,g)). In continuation, inhibition of miR-122-5p also eliminated the elevation in ROS level and the decrease in CAT, SOD, GSH-px activities induced by LPS (Figure 4(a,b)). MiR-122-5p inhibition decreased the generation of LPS-induced TNF-α, IL-6, IL-1β (Figure 4(e)). In addition, we found that LPS led to a reduction in Nrf-2 accumulation in the nucleus, accompanied by declined HO-1 and NQO-1 levels, but these results were reversed by miR-122-5p inhibition (Figure 4(c,d)). All findings indicate that inhibition of miR-122-5p prevents LPS-induced inflammatory response, oxidative stress, and apoptosis in cardiomyocytes, which may be involved in activation of the Nrf-2/HO-1 pathway.

### 3.4. GIT1 is a binding effector of miR-122-5p

**Figure 5.**
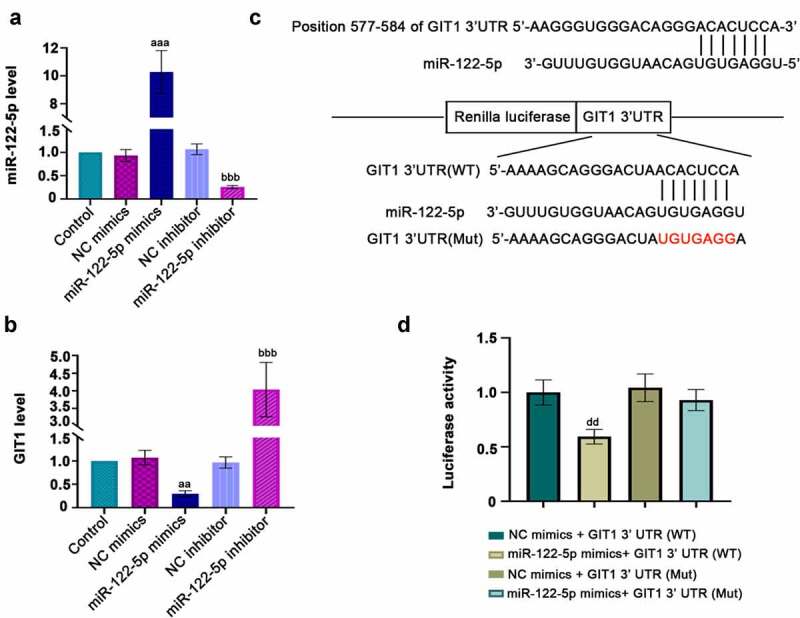
Potential downstream target gene of micro RNA miR-122-5p. H9c2 cells were transfected with NC mimics, miR-122-5p mimics, NC inhibitor and miR-122-5p inhibitor for 48 h. (a-b) The expression levels of miR-122-5p and G-protein-coupled receptor kinase interacting protein-1 (GIT1) were verified by real-time quantitative PCR (RT-qPCR) assay. (c) The predicted binding sites of miR-122-5p in the 3ʹ-UTR of GIT1, and the sequence information of miR-122-5p and GIT1 (wild- or mutant- type) was displayed. (d) Luciferase assay verified the correlation between miR-122-5p and GIT1. ^aa^*p* < 0.01, ^aaa^*p* < 0.001 versus NC mimics; ^bbb^*p* < 0.001 versus NC inhibitor; ^dd^*p* < 0.01 versus NC mimics + GIT1 3ʹUTR (WT)

To achieve the overexpression or knockdown of miR-122-5p, H9c2 cells were transfected with miR-122-5p mimics, or miR-122-5p inhibitor or their controls NC mimics and NC inhibitor. After 48 h of transfection, RT-qPCR was performed to verify the efficiency of transfection. It was shown that miR-122-5p expression was increased by miR-122-5p mimics, but it was decreased by miR-122-5p inhibitor (Figure 5(a)). Also, miR-122-5p overexpression significantly lowered GIT1 level, while miR-122-5p inhibition enhanced it (Figure 5(b)). To clarify the relationship between miR-122-5p and GIT1, the bioinformatics sites Targetscan and miRDB were utilized. Figure 5(c) showed the 3ʹUTR sequences of wild-type and mutant GIT1 and the potential seed pairing sites between miR-122-5p and GIT1. In parallel, luciferase reporter assay suggested that miR-122-5p mimics reduced the luciferase activity of GIT1 (WT) rather than GIT1 (Mut) (Figure 5(d)). Overall, we verify that GIT1 may be a target gene of miR-122-5p in myocardial cells.

### 3.5. Silencing GIT1 diminished the beneficial effect of miR-122-5p loss in LPS-induced cardiomyocyte injury

**Figure 6.**
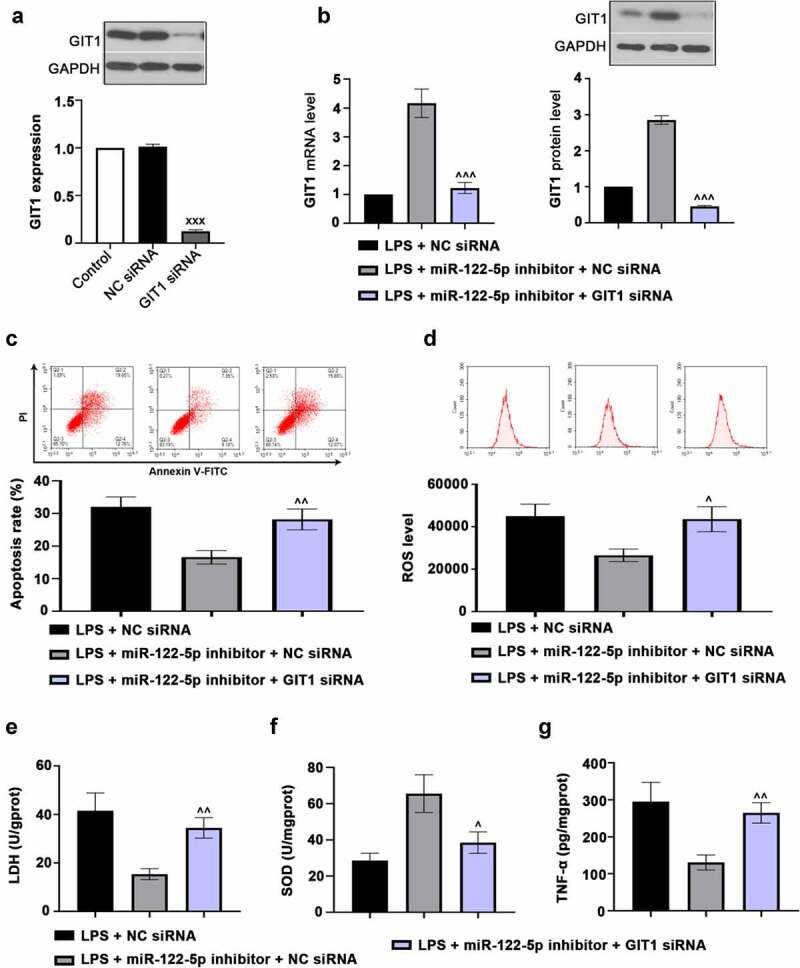
G-protein-coupled receptor kinase interacting protein-1 (GIT1) deficiency attenuates the effects of micro RNA miR-122-5p loss on myocardial injury. (a) H9c2 cells were transfected with GIT1 siRNA to downregulate GIT1 expression. (b) The cells were transfected with GIT1 siRNA and/or miR-122-5p inhibitor, and then induced by lipopolysaccharide (LPS). GIT1 expression at mRNA and protein levels was then measured using real-time quantitative PCR (RT-qPCR) or western blot. (c) Apoptosis of myocardial cells was analyzed by flow cytometry. (d) Reactive oxygen species (ROS) production was examined using flow cytometry. (e-g) The contents of lactate dehydrogenase (LDH), superoxide dismutase (SOD) and tumor necrosis factor alpha (TNF-α) were assessed by the enzyme-linked immunosorbent assay (ELISA) kits. ^XXX^*p* < 0.001 versus NC siRNA; ^^^*p* < 0.05, ^^^^*p* < 0.01, ^^^^^*p* < 0.001 versus LPS + miR-122-5p inhibitor + NC siRNA

**Figure 7.**
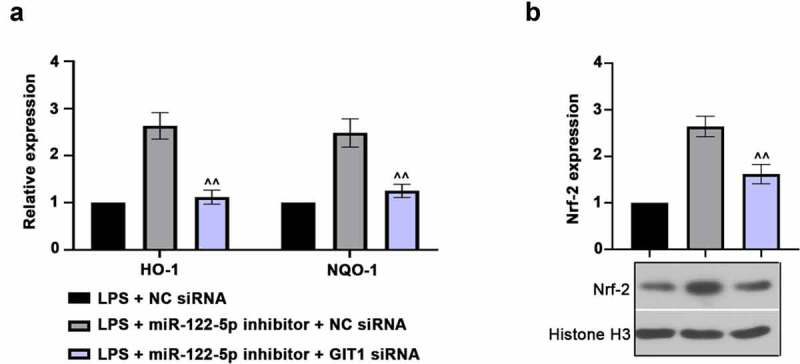
G-protein-coupled receptor kinase interacting protein-1 (GIT1) deficiency inhibits nuclear factor erythroid 2-related factor 2 (Nrf-2) activation. (a) Real-time quantitative PCR (RT-qPCR) assay was used to measure the heme oxygenase-1 (HO-1) and NAD(p)H: quinone oxidoreductase 1 (NQO-1) expression. (b) Nuclear Nrf-2 level was revealed using western blot analysis. ^^^^*p* < 0.01 versus LPS + miR-122-5p inhibitor + NC siRNA

To further explore whether miR-122-5p exhibited its role in sepsis-induced myocardial injury via miR-122-5p/GIT1 axis. We first knocked down GIT1 in H9c2 cells by transfection with GIT1 siRNA as displayed in Figure 6(a). Next, H9c2 cells were co-transfected with GIT1 siRNA and miR-122-5p inhibitor, and then subjected to LPS to confirm the function of miR-122-5p/GIT1 axis. It was found that GIT1 expression was reduced in LPS-induced myocardial cells (Figure 6(b)). Also, flow cytometry assays showed that GIT1 deficiency abolished the effect of miR-122-5p inhibition on cell apoptosis and ROS production (Figure 6(c,d)). Biochemical analysis demonstrated that GIT1 deficiency increased the LDH content and TNF-α level and reduced SOD activity (Figure 6(e-g)). In addition, we found that the effect of miR-122-5p inhibitor on Nrf-2/HO-1 pathway could be reversed by GIT1 deletion as reflected by reduced HO-1, NQO-1 and Nrf-2 expression levels (Figure 7(a,b)). Collectively, these data indicate that miR-122-5p inhibition may exert a protective role against septic myocardial injury via monitoring GIT1 expression and Nrf-2 activation.

## 4. Discussion

Myocardial injury occurs in the early stage of sepsis and is the main cause of death in patients with sepsis. Previous reports have revealed that miRNAs can serve as potential targets for the detection of sepsis [13,15]. In our study, we demonstrated that miR-122-5p expression was up-regulated in rat hearts and myocardial cells with LPS induction. With the *in vivo* and *in vitro* sepsis models, we found that inhibition of miR-122-5p alleviated LPS-induced myocardial injury, which was the first to reveal the function of miR-122-5p in septic heart injury. Also, GIT1 was down-regulated in response to sepsis, and it might be a target gene of miR-122-5p. Silencing GIT1 partially reversed the effect of miR-122-5p inhibition on myocardial dysregulation, which was possibly mediated by the Nrf-2/HO-1 signaling pathway.

Sepsis is considered to be a complicated biological process characterized by an overactive inflammatory reaction [27]. Increasing studies have shown that miRNAs play important roles in the regulation of immune response in sepsis [28]. In the current study, we demonstrated that miR-122-5p expression was elevated in response to sepsis, which was in line with those of a previous study [29]. However, little is known about the role of miR-122-5p in sepsis-induced myocardial injury and the underlying mechanisms. It is reported that the loss of miR-122-5p has anti-inflammatory effects in cardiovascular diseases [30]. Zhao et al. found that down-regulating miR-122-5p inhibits macrophage-related inflammation [18]. Consistent with these studies, our results suggested that inhibition of miR-122-5p reduced inflammatory cell infiltration and cTnI and LDH levels in the heart. Also, miR-122-5p inhibition significantly repressed the LPS-induced release of pro-inflammatory factors TNF-α, IL-6 and IL-1β in heart of septic rats and myocardial cells. These findings indicate the anti-inflammatory role of low miR-122-5p in septic myocardial injury. Moreover, oxidative stress is a process of pro-oxidant/antioxidant balance, which exhibits important roles in the pathogenesis of sepsis [31]. The involvement of miR-122-5p in oxidative stress has been the focus of research. Lu et al. revealed that miR-122-5p exaggerates LPS-induced oxidative stress in liver [32]. Song et al. showed that miR-122-5p inhibition prevents the promotion of angiotensin II–induced oxidative stress [33]. Our results showed that inhibiting miR-122-5p could decrease ROS generation and increase the activities of antioxidant enzymes CAT, SOD and GSH-px in septic rat and H9c2 cells. Thus, miR-122-5p inhibition has an antioxidant effect on myocardial injury with sepsis. Meanwhile, cell apoptosis is also a contributor to the progression of septic heart damage, and inhibition of myocardial cell apoptosis could attenuate heart injury [34]. It has shown that silencing miR-122-5p inhibits apoptosis of myocardial cells [35,36]. In accordance with our findings, miR-122-5p inhibition delayed LPS-triggered cell apoptosis *in vivo and in vitro*, along with reduced caspases-3 activity. Collectively, these findings indicate that miR-122-5p inhibition exerts anti-inflammatory, antioxidant and anti-apoptotic effects on sepsis-induced myocardial injury.

GIT1 is a multi-function scaffold protein that has been verified as an important regulator in the heart. Pang et al. revealed that silencing GIT1 promotes cardiomyocyte apoptosis and myocardial injury [22]. Our results demonstrated that GIT1 was a target gene of miR-122-5p and was low-expressed in sepsis. We speculated that the effect of miR-122-5p on sepsis may be associated with the modulation of GIT1. Moreover, Zhao et al. found that GIT1 possesses antioxidant and anti-inflammatory roles in LPS-induced macrophages [21]. In line with the previous studies, we confirmed that GIT1 deficiency partially reversed miR-122-5p inhibition-mediated protective effect on sepsis as evidenced by increased cell apoptosis, triggered ROS production, elevated LDH content, enhanced TNF-α level, and reduced SOD activity. These results demonstrated that the cardioprotective roles of miR-122-5p inhibition in sepsis-induced myocardial injury may be achieved by negatively regulating GIT1.

Nrf-2 is considered as a cytoprotective regulator, whose activation is associated with the attenuation of sepsis-induced heart injury [37]. We further analyzed whether the effect of miR-122-5p/GIT1 axis on sepsis is regulated by the Nrf-2 signaling pathway. Meng et al. reported that miR-122-5p loss attenuates LPS-induced liver injury through up-regulating Nrf-2 [38]. Qiu et al. found that miR-122-5p contributes to the down-regulation of HO-1, a downstream regulator of Nrf-2 [39]. Zhao et al. also revealed that GIT1 can activate Nrf-2 and reduce inflammatory response in LPS-induced macrophages [21]. As expected, we found that inhibition of miR-122-5p elevated the total and nuclear Nrf-2 levels, as well as its downstream HO-1 and NQO-1, which were reversed by GIT1 depletion. These findings further support the possibility that miR-122-5p inhibition-mediated protective effect against myocardial injury in sepsis may be modulated by the GIT-1-induced Nrf-2 signaling pathway. Although there are limitations in identifying the possible pathogenic mechanisms of myocardial injury in sepsis, the discovery of the Nrf-2 signaling pathway in relation to miR-122-5p/GIT1 axis contributes to our better understanding of the pathogenesis of septic myocardial injury.

## 5. Conclusion

This study provides further evidence for the functional role of miR-122-5p in the regulation of sepsis-induced myocardial injury. Inhibition of miR-122-5p effectively alleviates LPS-induced myocardial injury and inhibits inflammatory response, oxidative stress, and apoptosis by targeting GIT1, which is possibly associated with the Nrf-2/HO-1 pathway. Thereby, miR-122-5p may be a promising therapeutic target for patients with septic myocardial injury.

## Supplementary Material

Supplemental Material

## Data Availability

The datasets generated during and/or analyzed during this study are available from the corresponding author on reasonable request.

## Highlights

- Inhibition of miR-122-5p suppressed LPS-triggered myocardial injury.
- MiR-122-5p inhibition reduced inflammatory response, oxidative stress and apoptosis in heart.
- GIT1 was verified as a target of miR-122-5p.
- Nrf-2/HO-1 is involved in regulating miR-122-5p/GIT1-mediated cardioprotection.

## References

[1] Singer M, Deutschman CS, Seymour CW, et al. The third international consensus definitions for Sepsis and septic shock (Sepsis-3). Jama. 2016;315(8):801–810. 26903338 10.1001/jama.2016.0287 PMC4968574

[2] Weber GF, Chousterman BG, He S, et al. Interleukin-3 amplifies acute inflammation and is a potential therapeutic target in sepsis. Science (New York, NY). 2015;347(6227):1260–1265. 10.1126/science.aaa4268 PMC4376966 25766237

[3] Cohen J, Vincent JL, Adhikari NK, et al. Sepsis: a roadmap for future research. Lancet Infect Dis. 2015;15:581–614. 25932591 10.1016/S1473-3099(15)70112-X

[4] Bickler SW, De Maio A. Dysfunction of the innate immune system during sepsis: a call for research. Crit Care Med. 2013;41(1):364–365. 23269155 10.1097/CCM.0b013e318270e57b

[5] Cawcutt KA, Peters SG. Severe sepsis and septic shock: clinical overview and update on management. Mayo Clin Proc. 2014;89(11):1572–1578. 25444488 10.1016/j.mayocp.2014.07.009

[6] Kotecha A, Vallabhajosyula S, Coville HH, et al. Cardiorenal syndrome in sepsis: a narrative review. J Crit Care. 2018;43:122–127. 28881261 10.1016/j.jcrc.2017.08.044

[7] Annane D, Bellissant E, Cavaillon JM. Septic shock. Lancet. 2005;365(9453):63–78. 15639681 10.1016/S0140-6736(04)17667-8

[8] Court O, Kumar A, Parrillo JE, et al. Clinical review: myocardial depression in sepsis and septic shock. Crit Care. 2002;6(6):500–508. 12493071 10.1186/cc1822 PMC153435

[9] Ambros V. The functions of animal microRNAs. Nature. 2004;431(7006):350–355. 15372042 10.1038/nature02871

[10] Maute RL, Schneider C, Sumazin P, et al. tRNA-derived microRNA modulates proliferation and the DNA damage response and is down-regulated in B cell lymphoma. Proceedings of the National Academy of Sciences of the United States of America. 2013; 110(4):1404–1409. 10.1073/pnas.1206761110 PMC3557069 23297232

[11] Ambros V. microRNAs: tiny regulators with great potential. Cell. 2001;107(7):823–826. 11779458 10.1016/s0092-8674(01)00616-x

[12] Bartel DP. MicroRNAs: genomics, biogenesis, mechanism, and function. Cell. 2004;116(2):281–297. 14744438 10.1016/s0092-8674(04)00045-5

[13] Wang H, Bei Y, Shen S, et al. miR-21-3p controls sepsis-associated cardiac dysfunction via regulating SORBS2. J Mol Cell Cardiol. 2016;94:43–53. 27033308 10.1016/j.yjmcc.2016.03.014

[14] An R, Feng J, Xi C, et al. miR-146a attenuates Sepsis-induced myocardial dysfunction by suppressing IRAK1 and TRAF6 via targeting ErbB4 expression. Oxid Med Cell Longev. 2018;2018:7163057. 30224945 10.1155/2018/7163057 PMC6129849

[15] Zhu XG, Zhang TN, Wen R, et al. Overexpression of miR-150-5p alleviates apoptosis in Sepsis-induced myocardial depression. Biomed Res Int. 2020;2020:3023186. 32908879 10.1155/2020/3023186 PMC7477614

[16] Xu J, Feng Y, Jeyaram A, et al. Circulating plasma extracellular vesicles from septic mice induce inflammation via MicroRNA- and TLR7-dependent mechanisms. J Immunol. 2018;201(11):3392–3400. 30355788 10.4049/jimmunol.1801008 PMC6240609

[17] Zhang TN, Yang N, Goodwin JE, et al. Characterization of circular RNA and microRNA profiles in septic myocardial depression: a Lipopolysaccharide-induced rat septic shock model. Inflammation. 2019;42(6):1990–2002. 31332662 10.1007/s10753-019-01060-8

[18] Zhao Z, Zhong L, Li P, et al. Cholesterol impairs hepatocyte lysosomal function causing M1 polarization of macrophages via exosomal miR-122-5p. Exp Cell Res. 2020;387(1):111738. 31759057 10.1016/j.yexcr.2019.111738

[19] Aslan JE. Platelet Rho GTPase regulation in physiology and disease. Platelets. 2019;30(1):17–22. 29799302 10.1080/09537104.2018.1475632

[20] Zhang LQ, Zhao GZ, Xu XY, et al. Integrin-β1 regulates chondrocyte proliferation and apoptosis through the upregulation of GIT1 expression. Int J Mol Med. 2015;35(4):1074–1080. 25715677 10.3892/ijmm.2015.2114

[21] Zhao SJ, Liu H, Chen J, et al. Macrophage GIT1 contributes to bone regeneration by regulating inflammatory responses in an ERK/NRF2-dependent way. J Bone Miner Res: the official journal of the American Society for Bone and Mineral Research. 2020;35(10):2015–2031. 10.1002/jbmr.4099 PMC7689802 32460388

[22] Pang J, Xu X, Getman MR, et al. G protein coupled receptor kinase 2 interacting protein 1 (GIT1) is a novel regulator of mitochondrial biogenesis in heart. J Mol Cell Cardiol. 2011;51(5):769–776. 21756914 10.1016/j.yjmcc.2011.06.020 PMC3367479

[23] Yang N, Shi XL, Zhang BL, et al. The trend of β3-adrenergic receptor in the development of septic myocardial depression: a Lipopolysaccharide-induced rat septic shock model. Cardiology. 2018;139(4):234–244. 29566368 10.1159/000487126 PMC5969076

[24] Li P, Shen M, Gao F, et al. An antagomir to MicroRNA-106b-5p Ameliorates cerebral ischemia and reperfusion injury in rats via inhibiting apoptosis and oxidative stress. Mol Neurobiol. 2017;54(4):2901–2921. 27023223 10.1007/s12035-016-9842-1

[25] Liu X, Xiao J, Zhu H, et al. miR-222 is necessary for exercise-induced cardiac growth and protects against pathological cardiac remodeling. Cell Metab. 2015;21(4):584–595. 25863248 10.1016/j.cmet.2015.02.014 PMC4393846

[26] Kang W, Cheng Y, Zhou F, et al. Neuregulin‑1 protects cardiac function in septic rats through multiple targets based on endothelial cells. Int J Mol Med. 2019;44(4):1255–1266. 31432099 10.3892/ijmm.2019.4309 PMC6713419

[27] Huang M, Cai S, Su J. The pathogenesis of Sepsis and potential therapeutic targets. Int J Mol Sci. 2019;20(21):5376. 10.3390/ijms20215376 PMC6862039 31671729

[28] Mirna M, Paar V, Rezar R, et al. MicroRNAs in inflammatory heart diseases and Sepsis-induced cardiac dysfunction: a potential scope for the future? Cells. 2019;8(11):1352. 10.3390/cells8111352 PMC6912436 31671621

[29] Cortez-Dias N, Costa MC, Carrilho-Ferreira P, et al. Circulating miR-122-5p/miR-133b ratio is a specific early prognostic biomarker in acute myocardial infarction. Circ J: official journal of the Japanese Circulation Society. 2016;80(10):2183–2191. 27593229 10.1253/circj.CJ-16-0568

[30] Liu Y, Song JW, Lin JY, et al. Roles of MicroRNA-122 in cardiovascular fibrosis and related diseases. Cardiovasc Toxicol. 2020;20(5):463–473. 32856216 10.1007/s12012-020-09603-4 PMC7451782

[31] Lang MB, Segersvärd R, Grundsten M, et al. Management of alcohol use disorders in patients with chronic pancreatitis. JOP. 2012;13(6):654–659. 23183394 10.6092/1590-8577/1037

[32] Lu Z, Feng H, Shen X, et al. MiR-122-5p protects against acute lung injury via regulation of DUSP4/ERK signaling in pulmonary microvascular endothelial cells. Life Sci. 2020;256:117851. 32470454 10.1016/j.lfs.2020.117851

[33] Song JJ, Yang M, Liu Y, et al. MicroRNA-122 aggravates angiotensin II-mediated apoptosis and autophagy imbalance in rat aortic adventitial fibroblasts via the modulation of SIRT6-elabela-ACE2 signaling. Eur J Pharmacol. 2020;883:173374. 32682786 10.1016/j.ejphar.2020.173374 PMC7364171

[34] Li N, Zhou H, Wu H, et al. STING-IRF3 contributes to lipopolysaccharide-induced cardiac dysfunction, inflammation, apoptosis and pyroptosis by activating NLRP3. Redox Biol. 2019;24:101215. 31121492 10.1016/j.redox.2019.101215 PMC6529775

[35] Peng H, Luo Y, Ying Y. lncRNA XIST attenuates hypoxia-induced H9c2 cardiomyocyte injury by targeting the miR-122-5p/FOXP2 axis. Mol Cell Probes. 2020;50:101500. 31887421 10.1016/j.mcp.2019.101500

[36] Lin J, Zheng X. Salvianolate blocks apoptosis during myocardial infarction by down regulating miR-122-5p. Curr Neurovasc Res. 2017;14:323–329. 29076429 10.2174/1567202614666171026114630

[37] Rahim I, Sayed RK, Fernández-Ortiz M, et al. Melatonin alleviates sepsis-induced heart injury through activating the Nrf2 pathway and inhibiting the NLRP3 inflammasome. Naunyn-Schmiedeberg’s Archives of Pharmacology. 2021;394(2):261–277. 10.1007/s00210-020-01972-5 32936353

[38] Meng M, Zhang R, Han R, et al. The polysaccharides from the Grifola frondosa fruiting body prevent lipopolysaccharide/D-galactosamine-induced acute liver injury via the miR-122-Nrf2/ARE pathways. Food Funct. 2021;12(5):1973–1982. 33586729 10.1039/d0fo03327h

[39] Qiu L, Fan H, Jin W, et al. miR-122-induced down-regulation of HO-1 negatively affects miR-122-mediated suppression of HBV. Biochem Biophys Res Commun. 2010;398(4):771–777. 20633528 10.1016/j.bbrc.2010.07.021

