# Supplementary figures and images for: Inhibition of micro RNA miR-122-5p prevents lipopolysaccharide-induced myocardial injury by inhibiting oxidative stress, inflammation and apoptosis via targeting GIT1
Source: Bioengineered. 2021 May 18;12(1):1902–15. doi: 10.1080/21655979.2021.1926201 (PMC8806731; doi:10.1080/21655979.2021.1926201)

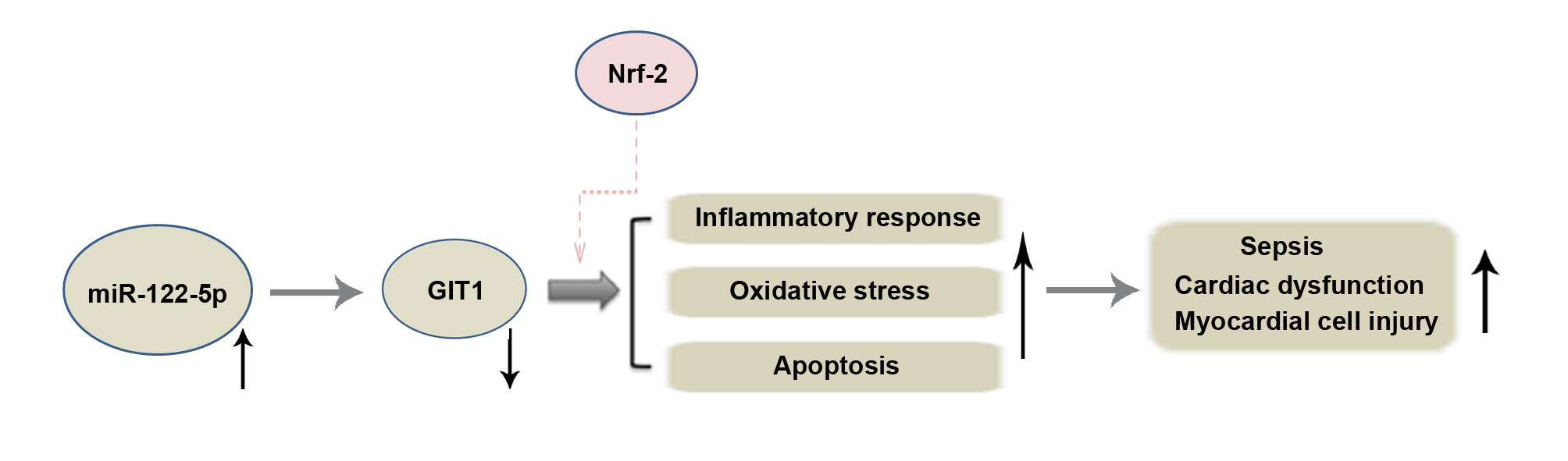

Supplement: Supplemental Material [file KBIE_A_1926201_SM6882.tif]
